# Effects of Lung Injury on Regional Aeration and Expiratory Time Constants: Insights From Four-Dimensional Computed Tomography Image Registration

**DOI:** 10.3389/fphys.2021.707119

**Published:** 2021-07-28

**Authors:** Jacob Herrmann, Sarah E. Gerard, Wei Shao, Yi Xin, Maurizio Cereda, Joseph M. Reinhardt, Gary E. Christensen, Eric A. Hoffman, David W. Kaczka

**Affiliations:** ^1^Department of Biomedical Engineering, Boston University, Boston, MA, United States; ^2^Department of Radiology, University of Iowa, Iowa City, IA, United States; ^3^Department of Radiology, Stanford University, Stanford, CA, United States; ^4^Department of Radiology, University of Pennsylvania, Philadelphia, PA, United States; ^5^Department of Anesthesiology and Critical Care, University of Pennsylvania, Philadelphia, PA, United States; ^6^Roy J. Carver Department of Biomedical Engineering, University of Iowa, Iowa City, IA, United States; ^7^Department of Electrical and Computer Engineering, University of Iowa, Iowa City, IA, United States; ^8^Department of Radiation Oncology, University of Iowa, Iowa City, IA, United States; ^9^Department of Internal Medicine, University of Iowa, Iowa City, IA, United States; ^10^Department of Anesthesia, University of Iowa, Iowa City, IA, United States

**Keywords:** mechanical ventilation, ventilator-induced lung injury, respiratory mechanics, computed tomography, image registration

## Abstract

**Rationale**: Intratidal changes in regional lung aeration, as assessed with dynamic four-dimensional computed tomography (CT; 4DCT), may indicate the processes of recruitment and derecruitment, thus portending atelectrauma during mechanical ventilation. In this study, we characterized the time constants associated with deaeration during the expiratory phase of pressure-controlled ventilation in pigs before and after acute lung injury using respiratory-gated 4DCT and image registration.

**Methods**: Eleven pigs were mechanically ventilated in pressure-controlled mode under baseline conditions and following an oleic acid model of acute lung injury. Dynamic 4DCT scans were acquired without interrupting ventilation. Automated segmentation of lung parenchyma was obtained by a convolutional neural network. Respiratory structures were aligned using 4D image registration. Exponential regression was performed on the time-varying CT density in each aligned voxel during exhalation, resulting in regional estimates of intratidal aeration change and deaeration time constants. Regressions were also performed for regional and total exhaled gas volume changes.

**Results**: Normally and poorly aerated lung regions demonstrated the largest median intratidal aeration changes during exhalation, compared to minimal changes within hyper- and non-aerated regions. Following lung injury, median time constants throughout normally aerated regions within each subject were greater than respective values for poorly aerated regions. However, parametric response mapping revealed an association between larger intratidal aeration changes and slower time constants. Lower aeration and faster time constants were observed for the dependent lung regions in the supine position. Regional gas volume changes exhibited faster time constants compared to regional density time constants, as well as better correspondence to total exhaled volume time constants.

**Conclusion**: Mechanical time constants based on exhaled gas volume underestimate regional aeration time constants. After lung injury, poorly aerated regions experience larger intratidal changes in aeration over shorter time scales compared to normally aerated regions. However, the largest intratidal aeration changes occur over the longest time scales within poorly aerated regions. These dynamic 4DCT imaging data provide supporting evidence for the susceptibility of poorly aerated regions to ventilator-induced lung injury, and for the functional benefits of short exhalation times during mechanical ventilation of injured lungs.

## Introduction

Repetitive recruitment and derecruitment of lung tissue during mechanical ventilation is associated with atelectrauma, a harmful process contributing to ventilator-induced lung injury and associated with progressively deteriorating overall condition (Slutsky and Ranieri, 2013; Gaver et al., 2020). Intratidal derecruitment occurs predominantly during exhalation, when the reduction of gas volume and distending pressure allows the collapse of atelectatic regions. Alveolar permeability, pulmonary edema, and surfactant dysfunction contribute to increased susceptibility to derecruitment in injured lungs (Gatto et al., 2004), such that atelectrauma is likely to occur in regions that are poorly aerated (Broche et al., 2017, 2019; Fardin et al., 2021). Regional distributions of intratidal recruitment and derecruitment have been inferred by comparing static or quasi-static computed tomography (CT) images acquired at end-expiration vs. end-inspiration (Crotti et al., 2001; Carvalho et al., 2008; Cereda et al., 2017). Clinical CT imaging cannot resolve structural details of individual alveoli and alveolar ducts, making it impossible to conclude whether an increase in CT density corresponds to partial derecruitment, uniform deflation without any derecruitment, or any combination thereof (Cereda et al., 2019). Nonetheless, progression of lung injury is associated with poor or unstable aeration in CT (Cereda et al., 2017; Xin et al., 2018). However, recruitment and derecruitment are not instantaneous responses to changes in transpulmonary pressure, but rather occur gradually over time, with heterogeneous rates of re-inflation and collapse (Bates and Irvin, 2002). Thus, to determine which regions of the lung may be at risk for atelectrauma, one must not only quantify how much aeration changes within a given region during exhalation, but also how quickly such deaeration occurs.

**Figure 1:**
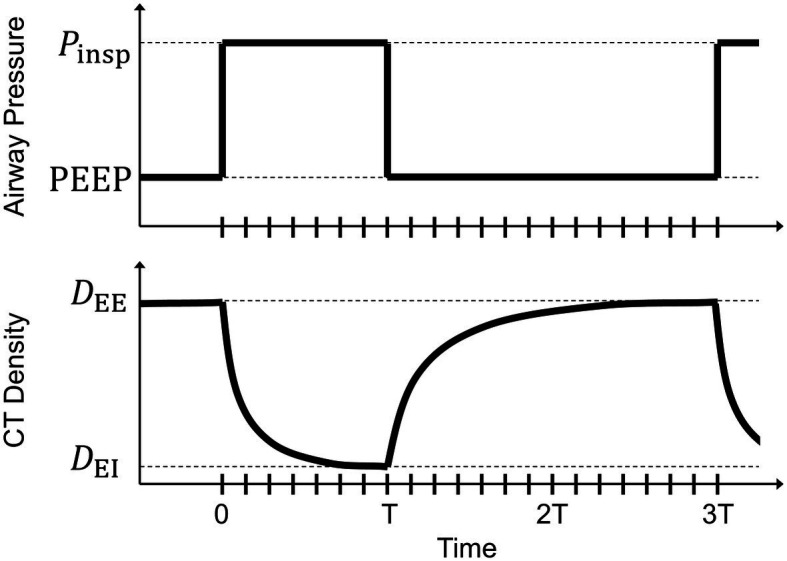
Schematic of a step change in airway pressure producing a decaying exponential response in voxel computed tomography (CT) density. At end-inspiration, airway pressure changes from inspiratory pressure (*P*_insp_) to positive end-expiratory pressure (PEEP) with an inspiratory:expiratory ratio of 1:2. Voxel CT density changes from an end-inspiratory level (*D*_EI_) to an end-expiratory level (*D*_EE_). Tick marks along the horizontal axis indicate the corresponding breath phases of retrospectively gated four-dimensional computed tomographic images. The duration of inspiration (T) is half that of exhalation (2 T).

Several lung-protective modalities are predicated on the maintenance of lung recruitment by shortening the time allowed for exhalation (Jain et al., 2016), with support from experimental evidence provided by *in vivo* microscopy at the alveolar level near the pleural surface (Gatto et al., 2004; Carney et al., 2005), dynamic CT (Neumann et al., 1998a,b; Markstaller et al., 2001), and synchrotron radiation phase-contrast imaging with sub-acinar spatial resolution (Fardin et al., 2021). Previous studies involving dynamic CT imaging have assumed exponential decay of regional aeration (i.e., changes in fraction of gas per unit volume of lung tissue, assessed by changes in regional CT density) in response to instantaneous changes in applied airway pressure (Figure 1). These studies demonstrated regional discrepancies in exhalation deaeration rates, but were limited in the ability to spatially localize these measurements.

The goal of this study was to quantify regional rates of lung deaeration during passive exhalation, using four-dimensional computed tomography (4DCT) dynamic imaging (Herrmann et al., 2017) and 4D image registration (Zhao et al., 2016; Herrmann et al., 2020a) to track localized aeration changes with high spatial resolution. In addition to assessment of aeration based on CT density, it is also possible to account for specific gas volume changes using an intensity-corrected Jacobian determinant, which is more strongly correlated to specific ventilation (Ding et al., 2012). In this study, we quantified regional CT density and gas volume time constants before and after acute lung injury in anesthetized porcine subjects. We also investigated potential factors that influence the distribution of these regional time constants, including location on the gravitational axis as well as regional aeration at end-inspiration, end-expiration, and predicted equilibrium. Our study therefore provides new insights into how the rate of tidal aeration changes occur within normally and poorly aerated regions during passive exhalation. Portions of these data were presented in abstract form at meetings of the American Society of Anesthesiologists (Kaczka et al., 2020) and the American Thoracic Society (Herrmann et al., 2020b).

## Materials and Methods

All experimental procedures were approved by the University of Iowa Institute for Animal Care and Use Committee (Protocol Number 5061428). Two other studies of respiratory mechanics, involving regional dynamic deformation (Herrmann et al., 2020a) and gas transport (Herrmann et al., 2021), were previously published using data collected from the subjects used in this study. Eleven pigs were used in this study, weighing between 9 and 13 kg.

Ventilation and CT imaging were performed before and after an acute lung injury induced by infusion of 0.08 cm^3^ kg^−1^ oleic acid into the internal jugular vein over 15 min. Maturation of lung injury was confirmed by a ratio of arterial oxygen tension to inspired oxygen fraction less than 300 mm Hg with at least 5 cm H_2_O of positive end-expiratory pressure (PEEP). Each subject was then mechanically ventilated in a pressure-controlled modality for 30 min, using a FabianHFO hybrid oscillator/ventilator (ACUTRONIC Medical Systems AG, Switzerland). During baseline conditions, *F*_i_O_2_ was set to 40%, but was increased to maintain *S*_p_O_2_ ≥ 90% following lung injury. Respiratory rate varied between 20 and 32 min^−1^, with inspiratory-to-expiratory (I:E) ratio of 1:2. Sampling frequency for the ventilator waveforms was 200 Hz. Ventilator driving pressure was adjusted to obtain arterial CO_2_ tension (*P*_a_CO_2_) in the target range of 30–60 mm Hg, while maintaining mean airway pressure (${\bar{P}}_{\mathrm{aw}}$) at 12 cm H_2_O. Each 30-min ventilation interval was followed by an arterial blood gas analysis and 4DCT scan sequence, without interrupting mechanical ventilation (Herrmann et al., 2017). Before each ventilation interval, a 30-second recruitment maneuver to 35 cm H_2_O of airway pressure was used to restore a control mechanical and physiological state. After completion of the experimental protocol, subjects were euthanized with an intravenous solution of pentobarbital sodium and phenytoin sodium (1 ml + 0.2 ml kg^−1^).

Computed tomography scans were acquired using a Siemens SOMATOM Force (Siemens Healthineers, Germany) in an axial scanning mode, with 5.76 cm of axial coverage and 0.6 mm slice thickness. Subjects were continuously scanned for 30 s at 80 kVp tube voltage and 150 mA tube current, with 250 ms scanner rotation period, yielding an extensive series of *x*-ray projection data. Projection data were then retrospectively binned according to both the scanner rotation angle and acquisition timing relative to the periodic ventilation cycle (Herrmann et al., 2017), resulting in a sequence of distinct sinograms each corresponding to a specific ventilation phase. Each sinogram sequence was then reconstructed with isotropic 0.6 mm spatial resolution to obtain a 4DCT image sequence, yielding between 13 and 21 volumetric images in each sequence (depending on sampling constraints imposed by the choice of respiratory rate). The temporal sampling frequency for 21-phase image sequences was 7 Hz during ventilation at 20 min^−1^, which is faster than the 4 Hz rotation frequency of the scanner. Each sequence was periodic in the temporal (i.e., phase) dimension, such that the choice of the “initial” image in the sequence was arbitrary. Voxels corresponding to spatial positions within the lungs were identified by a fully automated segmentation algorithm using a deep convolutional neural network (Gerard et al., 2018, 2020), generating a distinct lung mask for each image phase.

The periodic motion of respiratory structures was estimated using a deformable image registration technique, using four-dimensional cubic B-splines ensuring smoothness across both spatial and temporal dimensions (Metz et al., 2011). Fluctuations in CT voxel density due to variations in fractional gas content were compensated using a sum of squared tissue volume differences (SSTVD) similarity cost function (Gorbunova et al., 2008; Yin et al., 2009; Zhao et al., 2016), implemented in the Elastix library (Klein et al., 2010). After registration, the estimated transformations were used to deform images to align structures with a single, arbitrarily selected reference phase. The phase most closely aligned with the end of inspiration was manually identified by the lowest level of aeration according to time-varying histograms of CT intensity. End-expiratory phase was then determined according to the 1:2 I:E ratio for all subjects. The elapsed time since end-inspiration was determined for each expiratory phase according to the fixed time interval associated with each phase interval.

Before exponential regression, images were downsampled to 1.2 mm isotropic resolution, to reduce the influence of noise on parameter estimation. The time-varying intensity within each voxel during exhalation only was fit to an exponential model:

(1) $${\hat{I}}_{n}=D_{0}+\left(D_{\infty }-D_{0}\right)\;\cdot \;\;\left(1-e^{-t_{n}/\tau_{D}}\right)$$

where ${\hat{I}}_{n}$ is the spatially varying estimate of actual CT intensity, *I_n_*, at each ventilation phase *n* corresponding to elapsed expiratory time *t_n_*, and *D_0_*, *D_∞_*, and *τ_D_* are the spatially varying estimates of initial density, equilibrium density, and density time constant, respectively. Equilibrium density *D_∞_* is defined as the predicted value of CT intensity after a prolonged exhalation of infinite duration, i.e., as $t_{n}\to \infty$. Note that initial density and end-inspiratory density are the same, whereas the equilibrium density and end-expiratory density are not necessarily the same, especially if the duration of exhalation is longer than five times the time constant. Figure 1 illustrates the concept of exponential decay in voxel aeration following a step change in airway pressure. Parameters were estimated by minimizing the sum of squared residuals between model-predicted intensity ${\hat{I}}_{n}$ and actual image itensity *I_n_* for each ventilation phase *n*.

Regional time constants were estimated for changes in gas volume, by computing regressions for the specific air volume change by corrected Jacobian (SACJ; Ding et al., 2012) instead of density. The Jacobian determinant $\left|J_{n\to 0}\right|$ describes the ratiometric total volume change of a voxel between ventilation phase *n* and the end-inspiratory reference phase 0:

(2) $$\left|J_{n\to 0}\right|=\frac{V_{n}}{V_{0}}$$

Specific air volume change by corrected Jacobian adjusts the Jacobian determinant to account for changes in voxel intensity as well, assuming that gas and tissue exhibit CT intensities of −1,000 and 0 HU, respectively (Ding et al., 2012):

(3) $${\mathrm{SACJ}}_{n}=\left|J_{n\to 0}\right|\frac{I_{n}}{I_{0}}-1$$

Note that SACJ as defined is zero at end-inspiration, and negative at end-expiration if there is a loss of gas volume in the corresponding region.

The coefficient of determination and *F* statistic for each regression were computed to determine whether the exponential regression significantly contributed to prediction of variability. Voxels for which the *F* test yielded *p* > 0.05 were excluded from further analysis. Regional time constants were grouped and analyzed in several different ways. Time constants were grouped by aeration level using standard thresholds for CT intensity: hyper-aerated below −900 HU, normally aerated between −900 and −500 HU, poorly aerated between −500 and −100 HU, and non-aerated above −100 HU. Time constants were grouped by relative height in the gravitational field given by the position along the dorsal-ventral axis for subjects in the supine position. Time constants were grouped according to location on a parametric response map (PRM) corresponding to the initial and equilibrium densities of each voxel. Finally, time constants were grouped according to the nonequilibrated difference between end-expiratory density and predicted equilibrium density. For reference, an overall time constant for lung exhalation was obtained by applying Equation (1) to the exhaled gas volume measured at the proximal end of the endotracheal tube by the mechanical ventilator. Dynamic elastance was estimated by the quotient of driving pressure and tidal volume, assuming that inspiratory flow was nearly zero at end-inspiration.

## Results

Table 1 shows summarized ventilation parameters across all 11 subjects. The oleic acid injury model produced significantly lower ratios of alveolar oxygen tension to inspired oxygen fraction, meeting the criteria for severe (three subjects), moderate (seven subjects), and mild (one subject) acute respiratory distress syndome (ARDS). Injury was also associated with significantly higher dynamic elastance, respiratory rate, and inspiratory pressures. There was no significant difference in PEEP or tidal volume.

**Table 1:** Summarized parameters for ventilation and respiratory system mechanics, before and after lung injury (mean ± SD).

	Baseline	Injured
Positive end-expiratory pressure (cmH_2_O)	8.1 ± 1.5	7.7 ± 0.9
Inspiratory pressure (cmH_2_O)	20.8 ± 1.6	25.4 ± 3.3^*^
Tidal volume (ml kg^−1^)	9.1 ± 1.7	7.5 ± 2.4
P_a_O_2_:F_i_O_2_ ratio (mmHg)	481 ± 85	136 ± 56^*^
Dynamic elastance (cmH_2_O L^−1^)	143 ± 32	248 ± 59^*^

Asterisks indicate a statistically significant difference between baseline and injured conditions according to a paired samples t-test (^*^p < 0.001). P_a_O_2_:F_i_O_2_ ratio, quotient of arterial oxygen partial pressure and inspired oxygen fraction.

**Figure 2:**
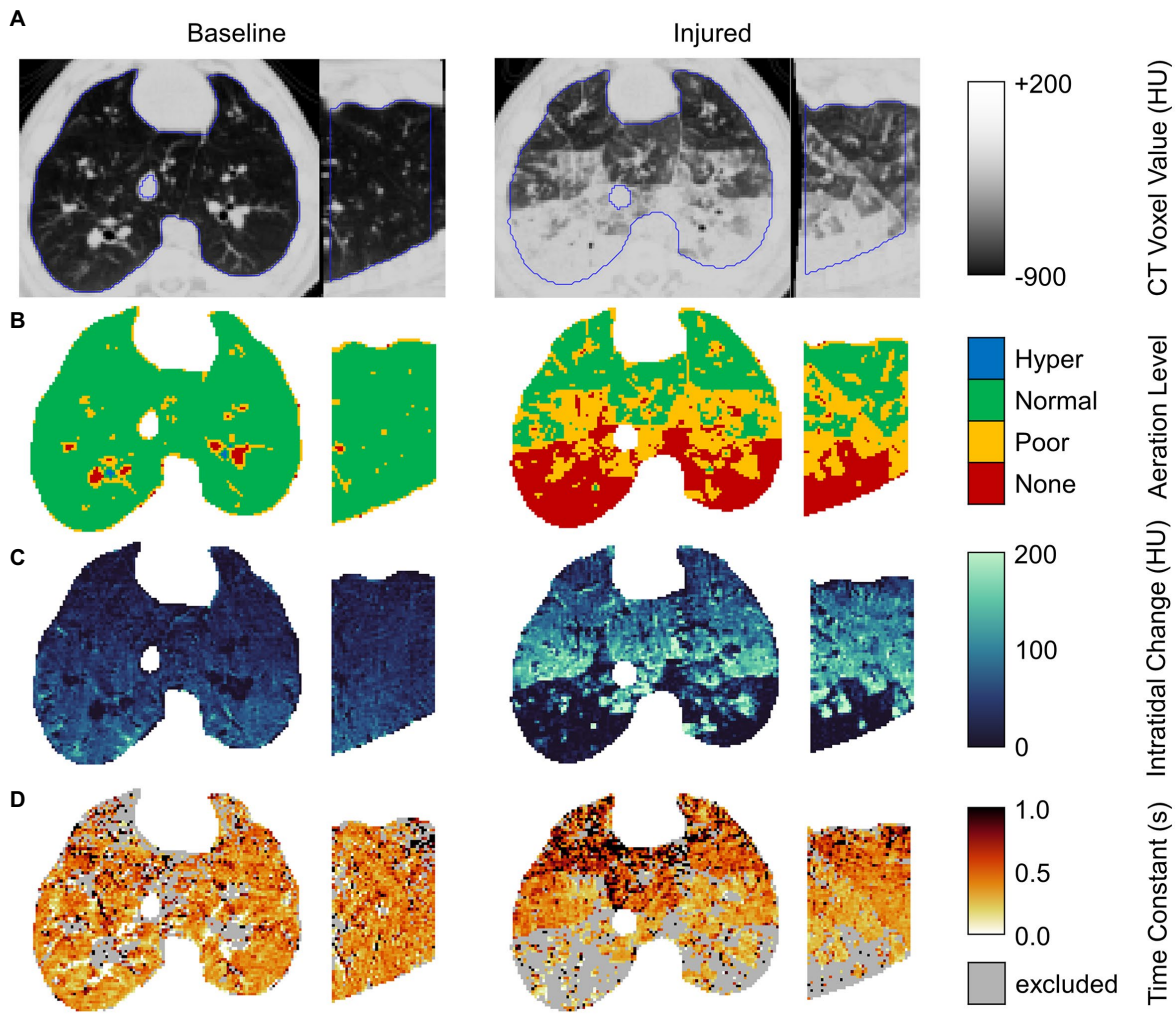
Example axial and sagittal views from images of a single representative subject before and after lung injury. From top to bottom, rows show **(A)** the end-expiratory CT image with lung segmentation (blue line); **(B)** end-expiratory aeration level; **(C)** the intratidal density change given by the difference between end-inspiratory and end-expiratory densities; and **(D)** the regional density time constant (excluding voxels for which exponential regression did not significantly contribute to prediction of variability at the 0.05 significance level).

Figure 2 shows regional aeration at end-inspiration, intratidal aeration change, and the time constant of expiratory deaeration in a representative subject before and after lung injury. Voxels were excluded if exponential regression did not significantly contribute to prediction of variability compared to the mean at the 0.05 significance level. The majority of excluded voxels were either hyper-aerated or non-aerated with little or no intratidal change in aeration, and thus relatively constant aeration throughout exhalation. Upon close visual inspection, such voxels often constituted airways, pulmonary vasculature, and areas of nonrecruiting atelectasis.

**Figure 3:**
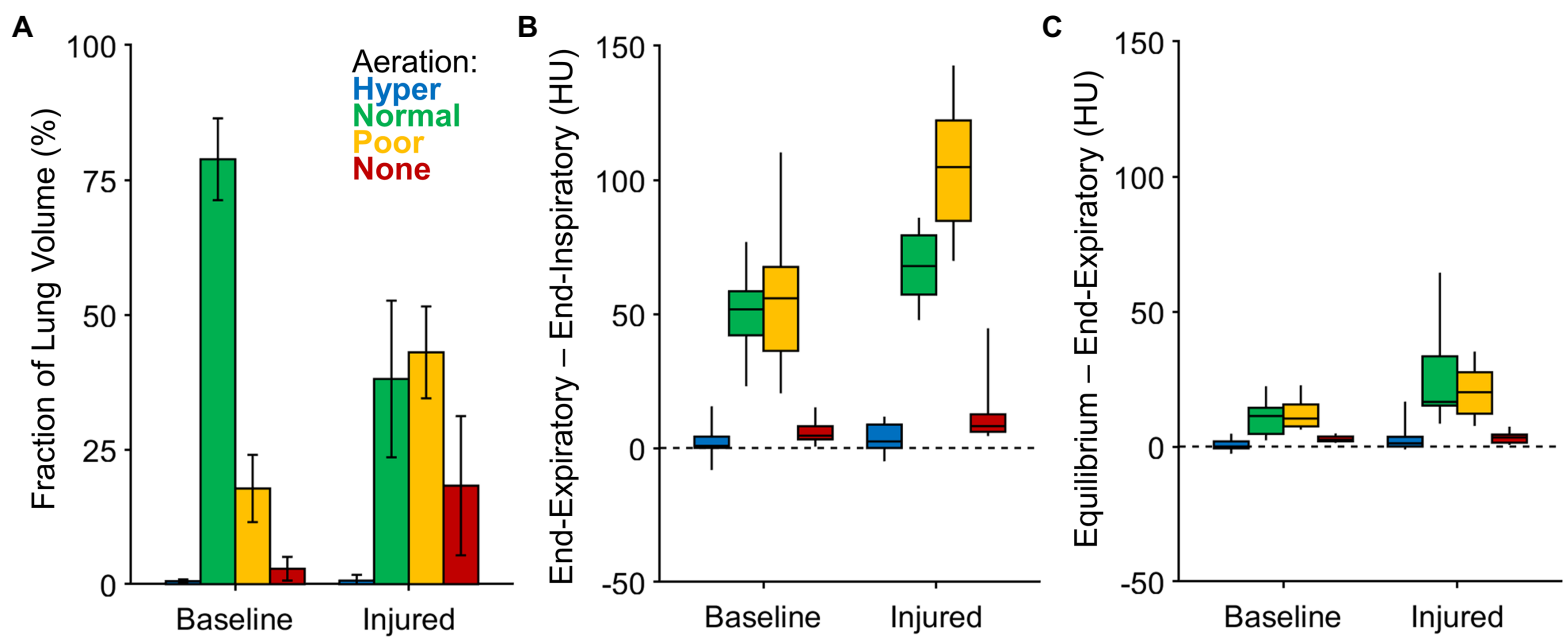
Aeration levels at end-expiration, end-inspiration, and predicted equilibrium. **(A)** The fraction of imaged lung volume at each end-expiratory aeration level. **(B)** The intratidal density change or the difference between end-expiratory density and end-inspiratory density, among voxels at each aeration level. **(C)** The nonequilibrated remaining density change or the difference between predicted equilibrium density and end-expiratory density, among voxels at each aeration level.

Figure 3A shows the average volume fractions of hyper-, normally, poorly, and non-aerated lung. Figures 3B,C show the intratidal change in density (i.e., the difference between end-expiratory and end-inspiratory densities), as well as the remaining nonequilibrated density change (i.e., the difference between predicted equilibrium density and end-expiratory density). The vast majority of intratidal variation in density occurred in regions that were normally or poorly aerated. Intratidal density change per voxel was largest in poorly aerated regions after lung injury. Overall, voxel densities equilibrated to within 20% of the expected change.

**Figure 4:**
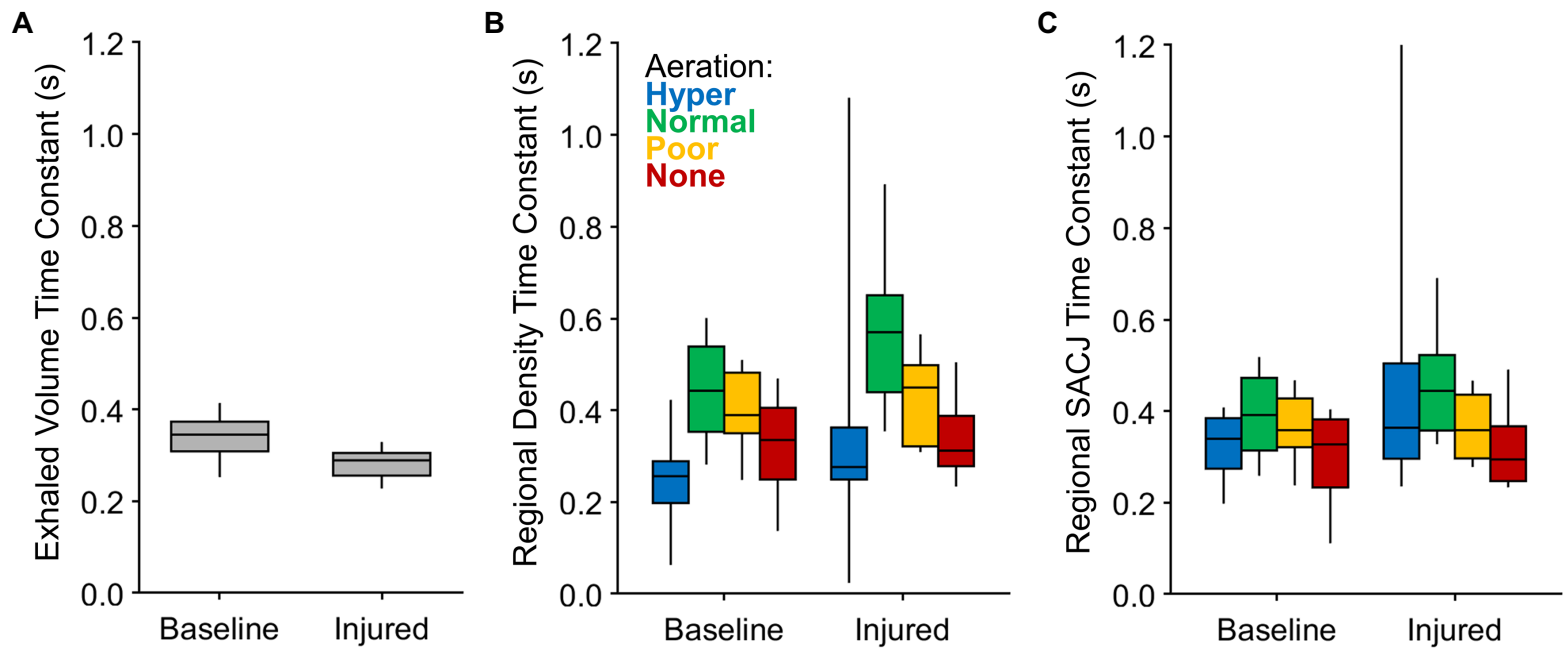
Expiratory time constants estimated for **(A)** the entire lung based on exponential regression of exhaled volume measured at the proximal end of the endotracheal tube; **(B)** lung regions based on exponential regression of density changes among voxels at each aeration level; and **(C)** lung regions based on exponential regression of specific air volume change by corrected Jacobian (SACJ) among voxels at each aeration level.

Figure 4 shows the regional density and SACJ time constants grouped according to aeration level, as well as the mechanical time constants for the entire respiratory system based on exhaled volume. Regional density time constants were typically larger (i.e., slower) than the overall time constants, but of the same order of magnitude. By contrast, the regional SACJ time constants were closer in magnitude to the overall time constant of the respiratory system. Regions with normal and poor aeration tended to exhibit slower regional time constants compared to hyper- and non-aerated regions. Although the overall time constants were reduced after lung injury, the regional density time constants were longer compared to baseline conditions.

**Figure 5:**
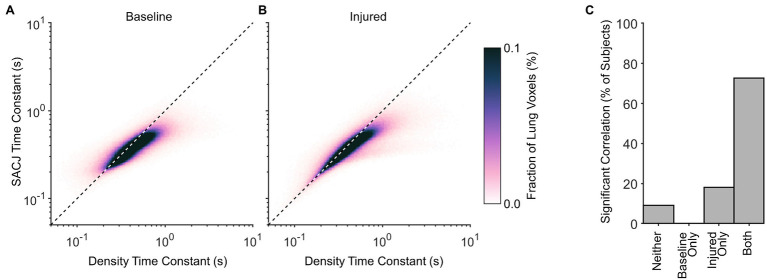
Correlation between regional time constants estimated for density changes and specific air volume change by corrected Jacobian (SACJ). Average probability density distributions are shown for **(A)** baseline and **(B)** injured conditions, including voxels for which both exponential regressions significantly predicted variability in the respective signals at the 0.05 significance level. Dashed line indicates identity. **(C)** The fraction of subjects exhibiting significant correlations between density and SACJ time constants before and/or after lung injury.

Figure 5 shows a strong correlation between regional density and SACJ time constants, with SACJ time constants consistently faster than density time constants in the same region. This trend was observed before and after lung injury.

**Figure 6:**
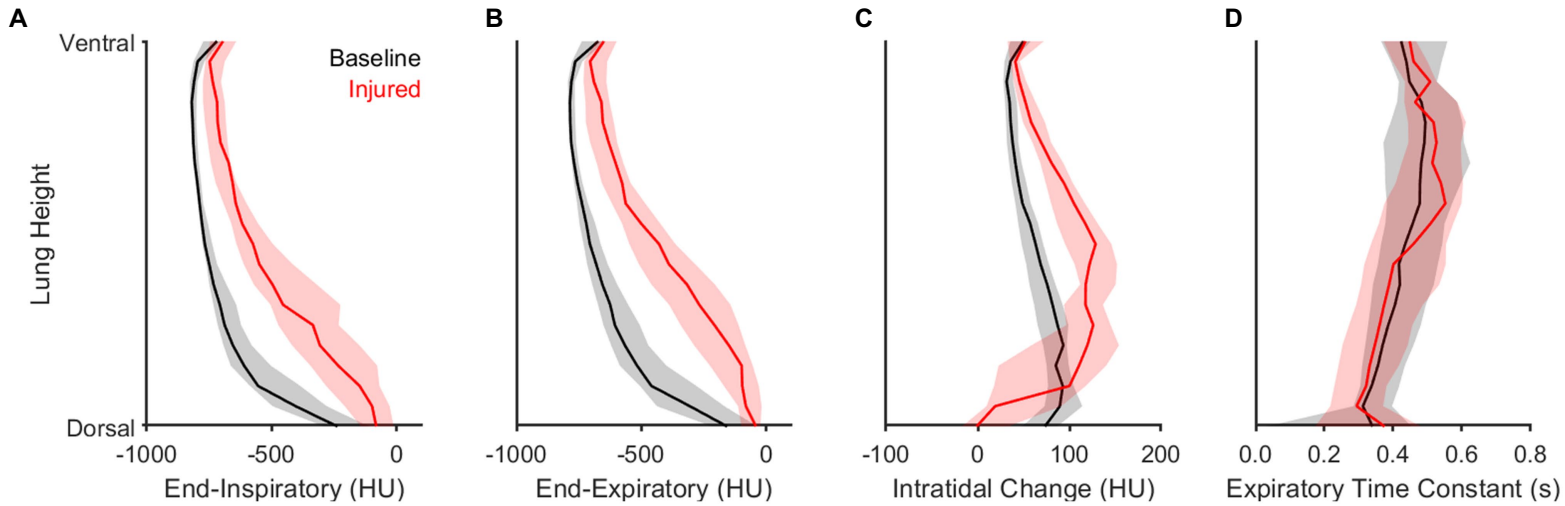
Influence of height and lung condition on regional intratidal density variation. Panels show median and interquartile range at each relative height level along the dorsal-ventral axis for **(A)** end-inspiratory density, **(B)** end-expiratory density, **(C)** the difference between end-expiratory and end-inspiratory densities, and **(D)** the density time constant.

Figure 6 highlights the contributions of gravitational stress and lung weight on regional aeration dynamics. Dorsal proximity in the supine position was associated with reduced aeration at end-expiration and end-inspiration, with a larger intratidal change in aeration, and with a faster density time constant. Lung injury resulted in a reduction of aeration at all height levels, especially in the dorsal lung. Lung injury was also associated with larger intratidal changes in aeration, particularly in the middle regions between ventral and dorsal portions. These middle regions generally exhibited poor aeration at end-expiration. Compared to baseline conditions, the density time constants were not different after lung injury for any height level.

**Figure 7:**
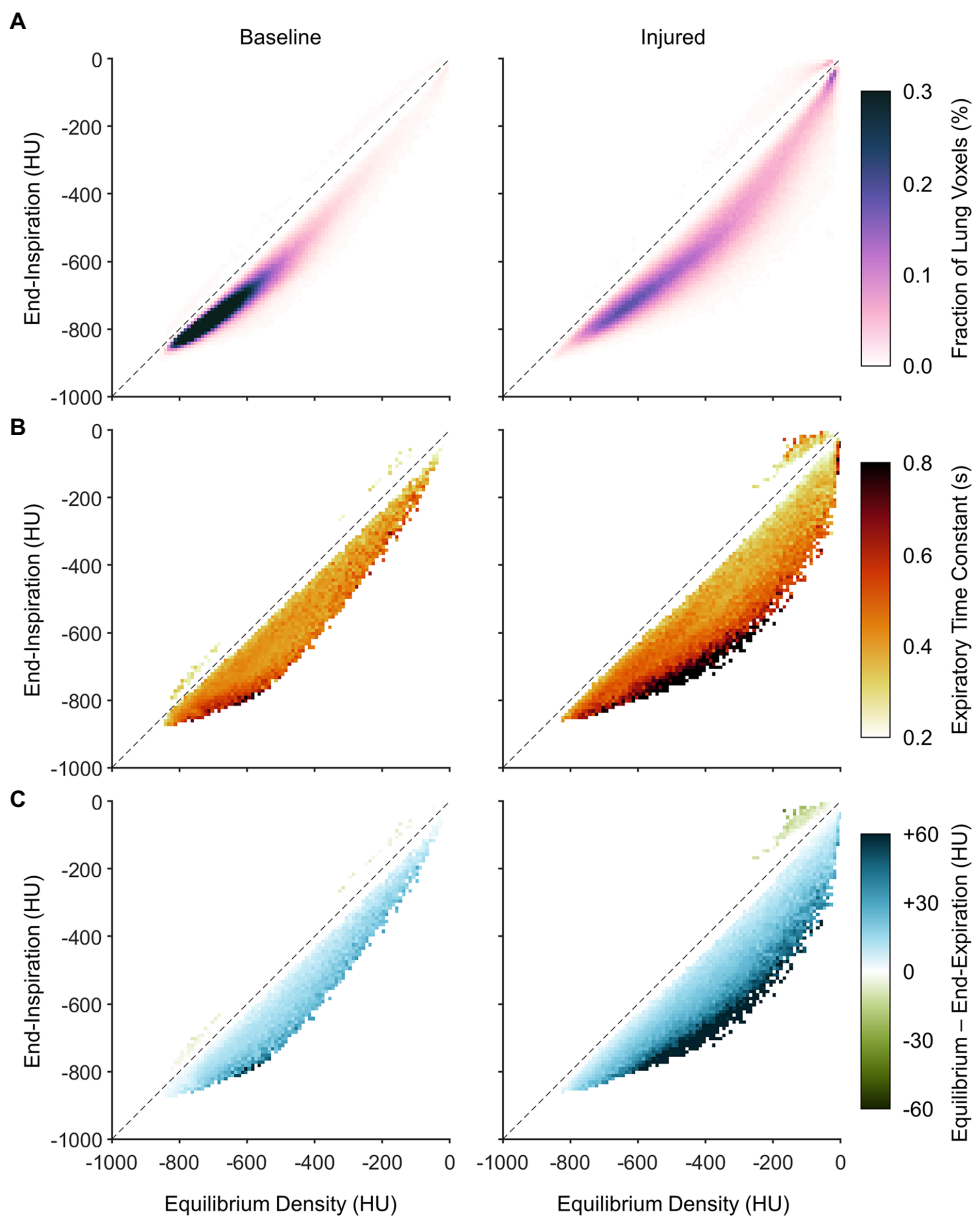
Distributions of dynamic aeration characteristics before and after lung injury with respect to location on the parametric response map (PRM), aggregated across all subjects. **(A)** Aggregate PRM showing the average probability density distribution of lung voxels with a given initial (or end-inspiratory) density and equilibrium density. Note that equilibrium density is not necessarily equal to end-expiratory density. The dashed line is the line of identity, indicating no change between end-inspiration and equilibrium. **(B)** Median density time constants, shown wherever at least half of the subjects exhibited at least five voxels each at the corresponding location of the PRM. **(C)** The median difference between equilibrium density and end-expiratory density, using the same inclusion criteria as **(B)**.

Figure 7A shows the average PRMs between end-expiratory and end-inspiratory density. The PRM in lung imaging typically depicts a two-dimensional histogram of lung voxels exhibiting each combination of end-expiratory and end-inspiratory density. In this figure, the predicted equilibrium density is used *in lieu* of the dynamic end-expiratory density, since PRMs are usually obtained quasi-statically *via* imaging during end-expiratory breathholds. Horizontal distance to the line of identity indicates the predicted density change between end-inspiration and equilibrium (i.e., after infinite exhalation duration). It is worth noting that a nonzero fraction of voxels was mapped above the line of identity, wherein density paradoxically decreases during exhalation. Figure 7B also shows how the distribution of density time constants depends on location within the PRM. In Figure 7B, the visualization represents not probability density but rather the median density time constant across subjects of voxels with end-expiratory and end-inspiratory aeration corresponding to that same location on the PRM. Data in this figure are aggregated from all subjects, such that any locations on the PRM without representation from at least half of all subjects were left blank. Regardless of lung condition, time constants were slower in regions that were normally aerated at both end-inspiration and end-expiration, compared to regions that were poorly aerated or non-aerated. However, there was also an effect of intratidal aeration change. Regions that were poorly aerated tended to have faster time constants when the predicted equilibrium change was small, and slower time constants when the predicted change was large. Figure 7C shows the nonequilibrated density change remaining at end-expiration (i.e., the difference between predicted equilibrium density and end-expiratory density). Similar to the observed distribution in Figure 7B, regions with larger predicted density changes also exhibited larger degrees of nonequilibration, as well as slower time constants.

**Figure 8:**
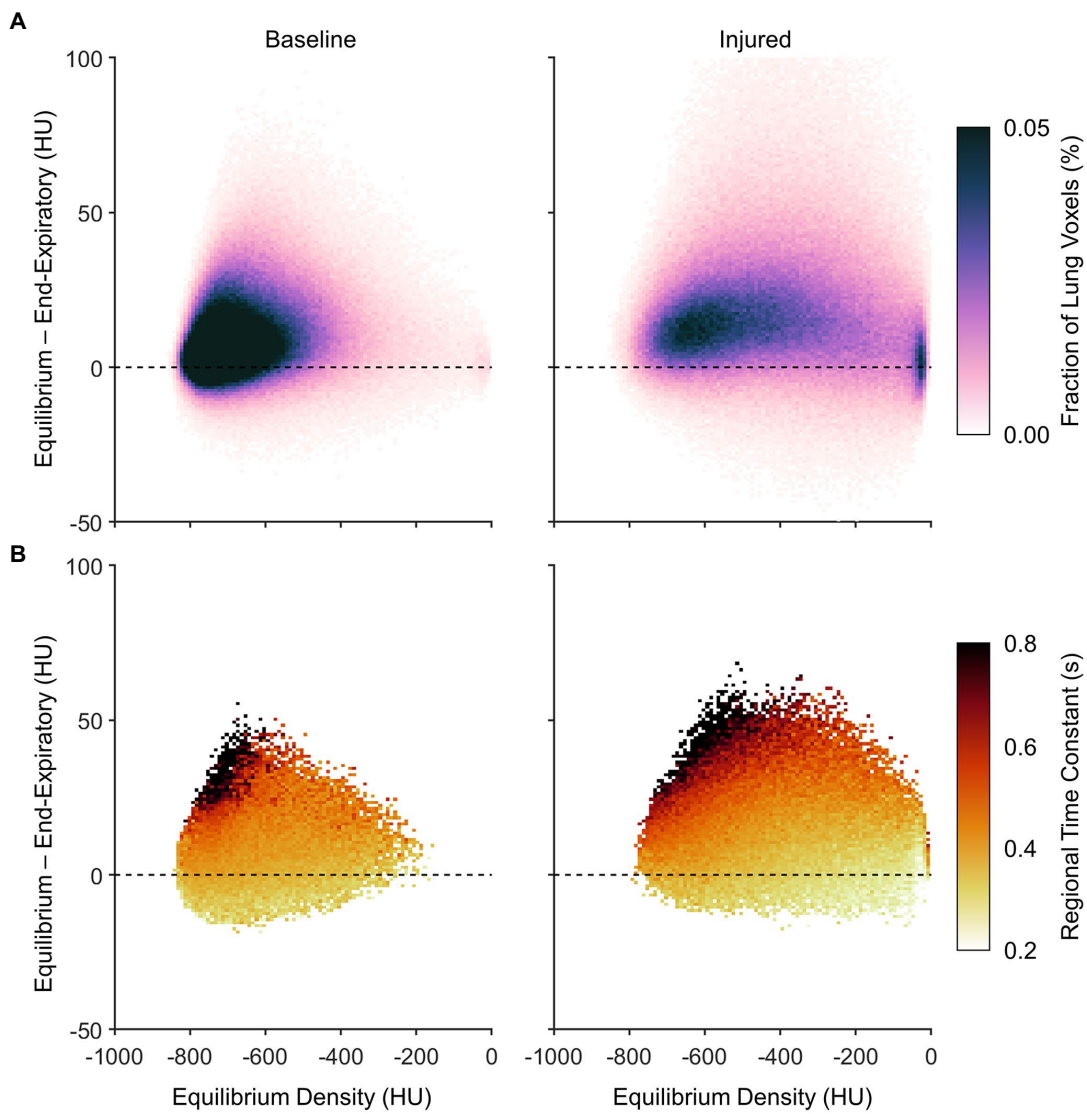
Characteristics of nonequilibrating density at end-expiration according to the difference between predicted equilibrium density and end-expiratory density, aggregated across all subjects. **(A)** Average probability density distribution, with increasing vertical distance from the dashed line indicating an increasing lack of convergence to the equilibrium density by end-expiration. **(B)** Median expiratory time constants, shown wherever at least half of the subjects exhibited at least five voxels each at the corresponding locations of **(A)**.

Figure 8A expresses the average probability density distribution in terms of the nonequilibrated density change, rather than the end-inspiratory density usually shown in PRMs as in Figure 7. This depiction emphasizes that injured regions with the largest degree of nonequilibrated density change also exhibited poor aeration at predicted equilibrium (i.e., between −500 and −100 HU). Figure 8B shows the corresponding median density time constants, again linking large nonequilibrated density change to slower time constants, especially in regions with normal aeration at predicted equilibrium (i.e., between −900 and −500 HU).

**Figure 9:**
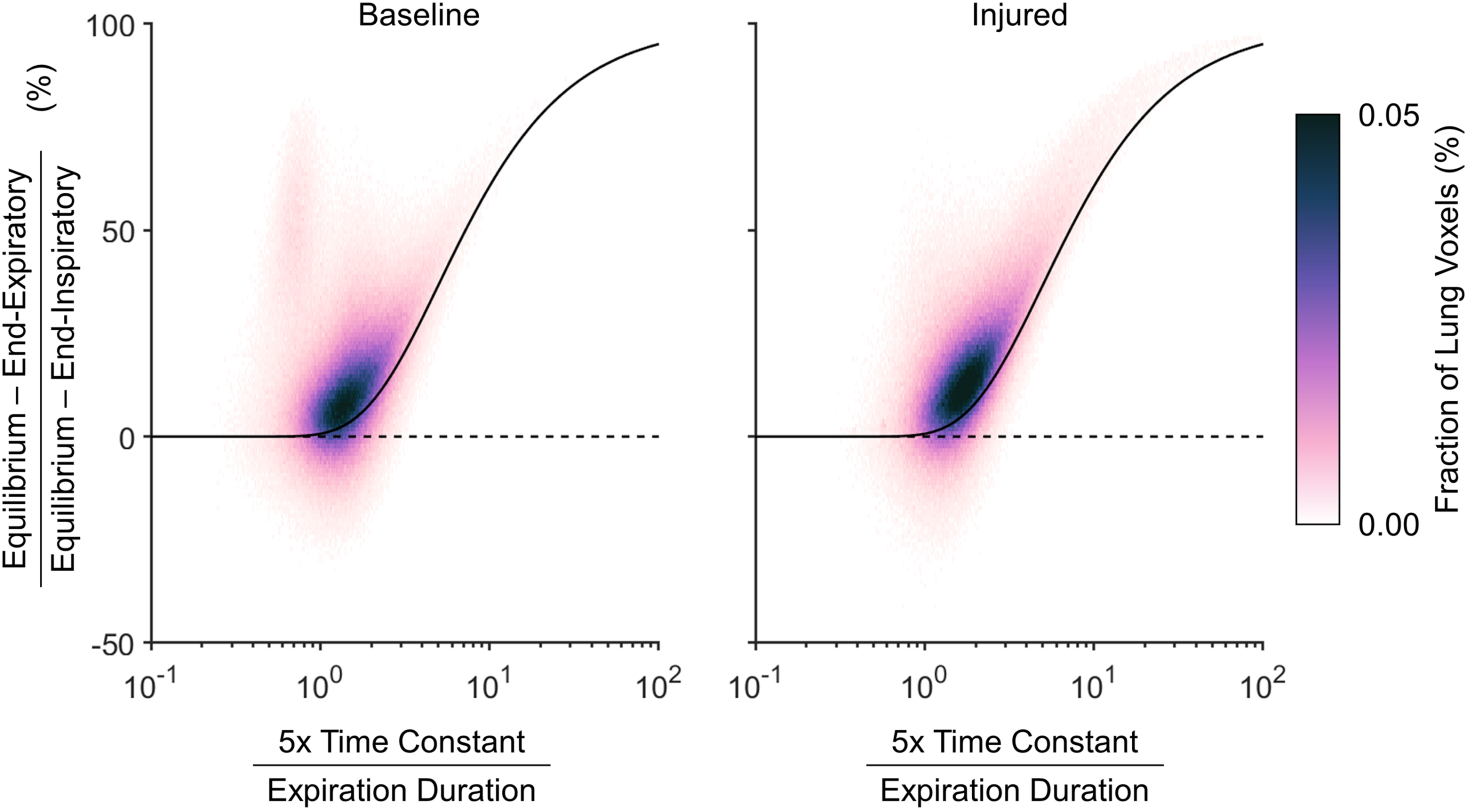
Relative nonequilibrated density change remaining at end-expiration, according to the difference between predicted equilibrium density and end-expiratory density normalized by the total expected density change from end-inspiration to equilibrium, shown with respect to the estimated density time constant normalized by the total duration allowed for exhalation. A voxel with an estimated density time constant less than one fifth of the exhalation duration is expected to converge to within 0.7%. The black line shows theoretical expected convergence for exponential decay.

Figure 9 shows the nonequilibrated density change as a normalized quantity, to represent the fractional convergence at end-expiration. The density time constant in this figure is also normalized by the duration of exhalation, which allows the resulting probability density distribution to be contextualized to the theoretical prediction for convergence of a single time constant exponential decay. The theoretical prediction provides a reliable lower bound for the nonequilibrated aeration changes. Regions with density time constant less than one fifth of the total exhalation duration tended to exhibit nearly complete equilibration.

**Figure 10:**
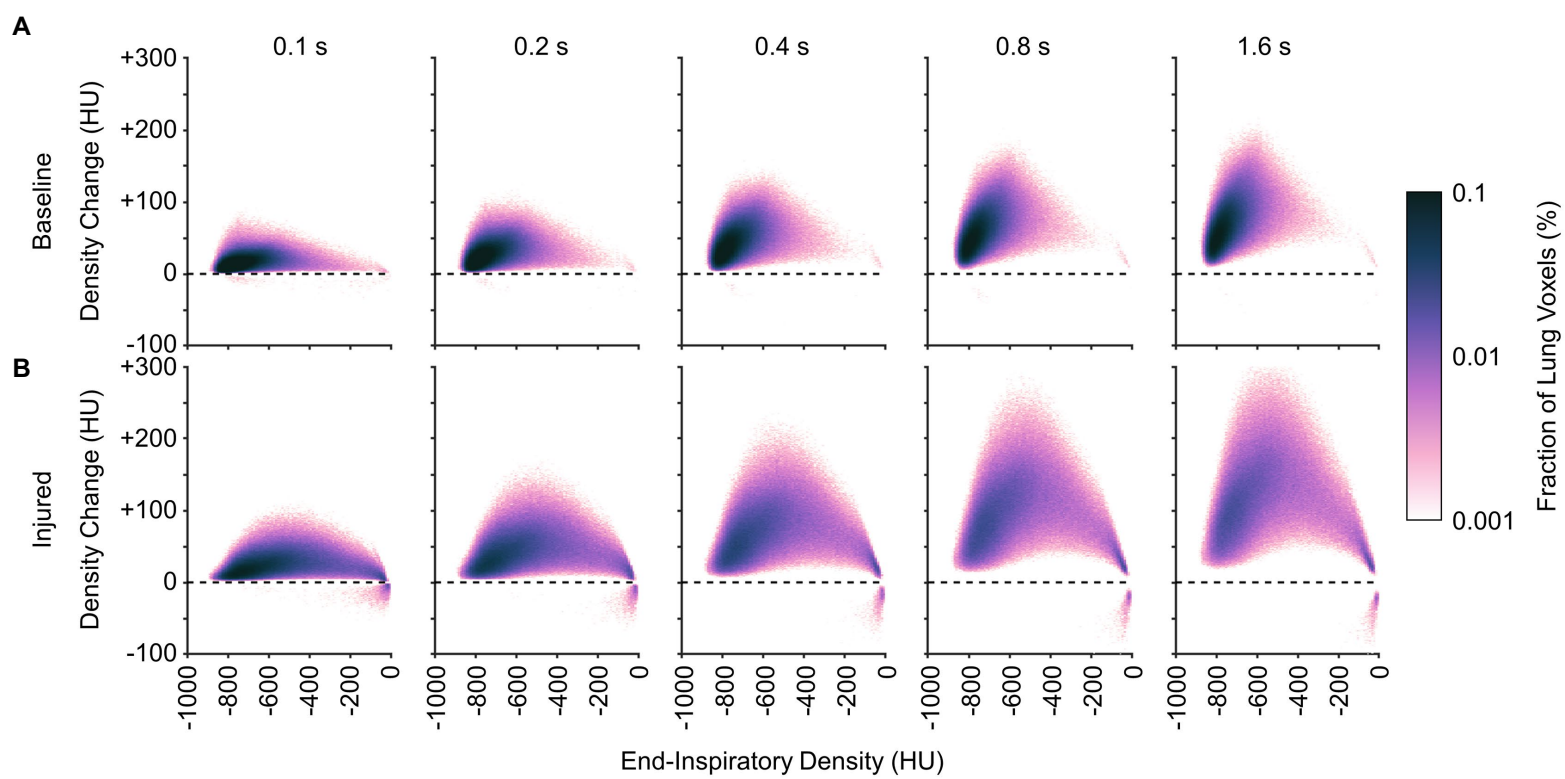
Average distributions of density change at different time points during exhalation **(A)** before and **(B)** after lung injury. Density change was estimated from exponential regression.

**Figure 11:**
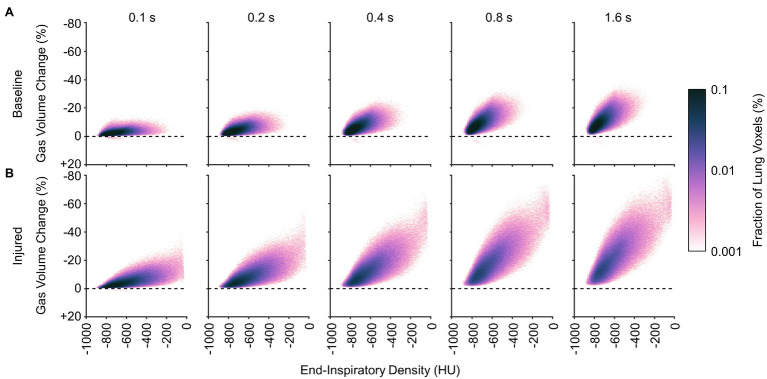
Average distributions of relative gas volume change at different time points during exhalation **(A)** before and **(B)** after lung injury.

Figures 10, 11 show the distributions of regional density change and gas volume change from end-inspiration as expiratory time increases. Regions with poor aeration exhibit the largest changes in density, and also the largest changes in gas volume. Changes in gas volume were magnified by low initial gas volume in regions that were poorly or non-aerated at end-inspiration. Regional gas volume reduced up to 33% under baseline conditions over longer exhalations, and up to 80% after lung injury. Restricting gas volume changes to less than 40% after injury would have required exhalation times less than 0.2 s according to the exponential regression.

## Discussion

This study presents a retrospective analysis of regional expiratory deaeration dynamics in mechanically ventilated pigs before and after lung injury. Previous studies of regional exhalation time constants using CT imaging have been limited to two-dimensional analyses and slower imaging speeds, permitting only coarse resolution of large spatial regions over relatively long expiratory durations (Neumann et al., 1998a,b; Markstaller et al., 2001). These studies were limited by a lack of anatomic specificity (i.e., lung tissue was moving through the image plane during acquisition), such that a given region of analysis in the image may have yielded different structures at each point in time. Additionally, slower sampling rates limited the faster time constants that could have been reliably estimated. In this study, we reconstructed volumetric sections of lung tissue with 5.6 cm axial coverage and temporal sampling rates of up to 21 images per breath. Together with 4DCT image registration, this study enabled a higher resolution analysis of local aeration changes over shorter time scales. Our use of image registration also enabled calculation of specific air volume changes by the so-called “corrected” Jacobian determinant (SACJ). The primary findings of this study include (1) a tendency for poorly aerated regions to exhibit large intratidal density changes and slow time constants; (2) a potential for preventing derecruitment of poorly aerated regions with shorter exhalation times; (3) a large discrepancy between the overall mechanical time constant of the respiratory system and regional deaeration time constants; (4) a potential for regions with slow time constants to remain nonequilibrated at end-expiration, especially in the injured lung; and (5) the gravitational dependence of regional time constants that is independent of lung injury.

It may be inferred from Figure 10 that shortening the allowable expiratory time during mechanical ventilation offers protection against derecruitment to regions of “unstable inflation,” or poor aeration. If instead an extended exhalation time is allowed, these poorly aerated regions at end-inspiration experience the greatest change in density, suggesting increased proclivity for collapse during exhalation. It should be noted that the same change in density has a larger effect on poorly aerated compared to normally aerated regions. The relative change in gas volume is amplified by the lower initial gas volume in poorly aerated regions. For example, normally aerated regions at end-expiration are less susceptible to derecruitment, whereas poorly aerated regions may not tolerate similar degrees of deaeration. Figure 11 highlights this discrepancy, with up to 80% loss of gas volume in poorly aerated regions of the injured lung during prolonged exhalation, compared to just 33% at baseline. For our lung injury model, ensuring a comparable upper limit of relative gas volume change requires a reduction of the exhalation duration to 0.2 s or shorter. There are also wide ranges of time constants in poorly aerated regions (Figure 8), with an association between slower time constants and larger changes in CT density (Figure 7). Thus, limiting exhalation time prevents the largest changes in density from occurring, perhaps providing more stability to poorly aerated regions.

The mechanical time constant for the entire respiratory system, as estimated by exponential regression of exhaled gas volume, was 10–50% faster compared to the regional density time constants, as estimated by exponential regression of CT density (Figure 4). Neumann et al. (1998a) also reported lower mechanical time constants (estimated by the product of respiratory system resistance and compliance) compared to CT density changes. The product of average resistance and compliance in their study was approximately 0.4 s both before and after oleic acid injury, compared to time constants ranging 0.7–1.4 s based on CT density changes (Neumann et al., 1998a). Furthermore, these authors found no correlation between CT density time constants and mechanical time constants, reasoning that extraneous mechanisms of density change (e.g., blood volume fluctuations; Porra et al., 2017) confound a meaningful relationship between overall mechanical time constants and regional CT density time constants (Neumann et al., 1998a).

The specific air volume change by corrected Jacobian (SACJ) utilizes an intensity-corrected Jacobian determinant to account for changes in gas volume only, yielding strong correlations to specific ventilation, at least as estimated by xenon-CT imaging (Ding et al., 2012). We computed exponential regression of regional SACJ variation over time, in an attempt to determine whether the regional SACJ time constants provided a better correspondence to the overall mechanical time constant of the entire respiratory system. Indeed, the discrepancy between overall and regional time constants was reduced using SACJ instead of CT density (Figure 3). This finding demonstrates that the rate of gas volume change is consistently faster than the rate of aeration change (Figure 5), both globally and regionally. This suggests that increases in regional tissue volume, due to blood influx and/or fluid accumulation, occur on a slower time scale compared to corresponding reductions in gas volume. It is worth noting that gas exchange relies on gas volume change specifically (i.e., the turnover of alveolar gas), as opposed to the CT density change (which reflects the relative amounts of tissue vs. gas in a voxel). Therefore, the observation that gas volume changes occur faster than density changes provides some justification for the efficacy of lung-protective strategies with short exhalation: enough time for alveolar gas turnover but not enough time for alveolar collapse (Bates et al., 2020). Although relative gas volume changes estimated by SACJ may be more appropriate for representation of the regional mechanical response, the primary objective of our study was to evaluate the dynamics of regional CT density instead of gas volume, since regional density is associated with atelectasis (Gatto et al., 2004; Broche et al., 2017; Fardin et al., 2021) and atelectrauma (Cereda et al., 2017). It is therefore important to consider that the mechanical time constant of the respiratory system, which can be easily estimated at the bedside during mechanical ventilation, may substantially underestimate regional exhalation time constants. Electrical impedance tomography may offer a low-resolution bedside alternative for measurement of aeration dynamics (Karagiannidis et al., 2018; Victor et al., 2019).

Cereda et al. (2017) identified locations on the PRM associated with the highest risk of injury progression and complete loss of aeration over the course of multiple hours. These high risk locations were characterized by voxels with “unstable inflation,” including those normally or poorly aerated at end-inspiration, but poorly or non-aerated at end-expiration. Several ventilatory strategies may reduce the fraction of lung operating in the high risk category, such as prone positioning (Xin et al., 2018) and use of PEEP. However, within the high risk PRM locations, there is still great variability of the dynamic characteristics of aeration change from end-inspiration to end-expiration. The 4DCT dynamic approach used in our study may supplement risk categorization based solely on end-inspiratory and end-expiratory densities, by contributing new information about precisely how density changes in each voxel between two endpoints. Importantly, our approach highlights the benefit of using short expiratory times as an alternative lung-protective strategy, to prevent derecruitment of high-risk lung regions. Such strategies include inverse ratio ventilation, airway pressure release ventilation, or time-controlled adaptive ventilation (Nieman et al., 2020).

Quasi-static imaging during end-expiratory breathholds likely overestimates the extent of aeration loss. The lung exhibits a wide distribution of region time constants, with some regions equilibrating over 10-fold longer time scales than others (Figure 4). Mechanical nonequilibration may contribute substantially to the measurement of regional aeration. Thus dynamic CT imaging, without interruption of mechanical ventilation, may yield more clinically relevant information. Even if gas flow is completely occluded, intrapulmonary gas redistribution and pendelluft may still affect the measurement of regional aeration, as well as time-dependent changes in regional recruitment (Bates and Irvin, 2002). Decreasing CT density during exhalation, evidenced in Figure 7, is a counterintuitive phenomenon that may indicate out-of-phase gas redistribution (Kaczka et al., 2011; Perchiazzi et al., 2014).

### Limitations

Our particular CT reconstruction technique relies on an assumption of temporal periodicity in the motion of thoracic structures (Herrmann et al., 2017). Asynchronous motion (e.g., due to cardiac contractions) may produce motion artifact and blurring. Furthermore, lung recruitment and derecruitment at the microscale exhibit irregularity over time (Broche et al., 2017), such that breath-to-breath variability of regional density may not be periodic. In our study, such irregular density variations are averaged over 30 s of CT scanning. The limited axial field-of-view provided by this imaging technique encompasses only a portion of the total lung volume. Apical and basal lung regions may exhibit different aeration responses. In addition, mean airway pressure was explicitly controlled in this study rather than PEEP, although all PEEP settings were between 5 and 10 cm H_2_O (Table 1). Neumann et al. (1998a) report faster time constants in an oleic acid model of lung injury when subjects were allowed to exhale to atmospheric pressure compared to 5 cm H_2_O PEEP yet no consistent difference in response to different PEEP levels greater than 10 cm H_2_O (Neumann et al., 1998b), highlighting the nonlinearity of respiratory system mechanics during passive exhalation.

We also did not determine the inspiratory time constants in our study. Given the 1:2 I:E ratio used in our study, only seven sequential images during inspiration could be reconstructed – potentially too few time points to ensure reliable regression especially in regions with longer time constants. Neumann et al. (1998a) also report faster time constants in pigs with an oleic acid model of lung injury during exhalation compared to inspiration, reasoning that the injured lung may tend to derecruit faster than it recruits.

Finally, our model for ARDS relied on oleic acid infusion into the central venous circulation, which mimics certain features of the fat emboli syndrome (Ballard-Croft et al., 2012). While the exact mechanism of injury from oleic acid remains elusive, it is associated with increased vascular permeability, as well as patchy, heterogenous edema in the airspaces and interstitium (Wang et al., 2008). Neumann et al. (1998a) noted differences in regional time constants among the oleic acid, saline lavage, and endotoxin models of lung injury. Unfortunately, CT imaging alone cannot distinguish between intraalveolar vs. interstitial edema in the poorly aerated regions we identified, at least for the spatial resolution of our scanner. Accordingly, one should exercise caution in generalizing our results in a small animal model of porcine oleic acid injury to mechanically ventilated humans with the acute respiratory distress syndrome. The underlying etiology of respiratory failure may yield differences in regional lung recruitability and dynamic behavior, as well as differences in noninjurious ventilatory approaches.

### Conclusion

Mechanical time constants for the entire respiratory system based on exhaled gas volume may underestimate the regional time constants of deaeration based on 4DCT imaging. After lung injury, poorly aerated regions tend to experience larger intratidal changes in aeration over shorter time scales compared to normally aerated regions. However, within these poorly aerated regions, the largest intratidal aeration changes occur over the longest time scales. These dynamic imaging results provide supporting evidence for the susceptibility of poorly aerated regions to ventilator-induced lung injury, and for the protective benefits of short exhalation times during mechanical ventilation of acutely injured lungs.

## Data Availability Statement

The raw data supporting the conclusions of this article will be made available by the authors, without undue reservation.

## Ethics Statement

The animal study was reviewed and approved by University of Iowa Institute for Animal Care and Use Committee.

## Author Contributions

JH and DK conceived the study, collected the data, and wrote the manuscript. JH, SG, WS, YX, MC, JR, GC, EH, and DK analyzed the data and revised the manuscript. All authors contributed to the article and approved the submitted version.

## Conflict of Interest

JH and DK are co-founders and shareholders of OscillaVent, Inc. and consultants for ZOLL Medical Corporation. JR and EH are co-founders and shareholders of VIDA Diagnostics, Inc., and GC is paid licensing fees from VIDA Diagnostics, Inc.

## Publisher’s Note

All claims expressed in this article are solely those of the authors and do not necessarily represent those of their affiliated organizations, or those of the publisher, the editors and the reviewers. Any product that may be evaluated in this article, or claim that may be made by its manufacturer, is not guaranteed or endorsed by the publisher.
